# Comparison of Artificial Intelligence Models and Human Experts in Managing Dyslipidemia: Assessment of Adherence to Clinical Guidelines

**DOI:** 10.7759/cureus.91363

**Published:** 2025-08-31

**Authors:** Mete Ucdal, Karya Yurtsever, Pinar Yildiz, Aysen Akalin, Kadir Ugur Mert, Gulay S Guven

**Affiliations:** 1 Department of Internal Medicine, Hacettepe University Faculty of Medicine, Ankara, TUR; 2 Department of Internal Medicine, Faculty of Medicine, Eskişehir Osmangazi University, Eskişehir, TUR; 3 Department of Endocrinology, Faculty of Medicine, Eskişehir Osmangazi University, Eskişehir, TUR; 4 Department of Cardiology, Faculty of Medicine, Eskişehir Osmangazi University, Eskişehir, TUR

**Keywords:** artificial intelligence, cardiovascular risk assessment, clinical guidelines, dyslipidemia, lipid management

## Abstract

Objective

The objective of this study is to compare guideline adherence between artificial intelligence (AI) models (Claude-3 (Anthropic, San Francisco, CA), DeepSeek-V2 (DeepSeek, Hangzhou, China), GPT-4 (OpenAI, San Francisco, CA)) and human experts in dyslipidemia management using standardized clinical scenarios based on 2019 European Society of Cardiology (ESC)/European Atherosclerosis Society (EAS) and 2021 ESC prevention guidelines. The study employed a comprehensive evaluation framework to capture the holistic nature of dyslipidemia management across multiple interconnected domains.

Methods

Thirty fictitious but clinically representative cases were developed by lipid specialists across five domains: cardiovascular risk assessment, lipid management, lifestyle modifications, pharmacotherapy, and special populations. This broad scope was deliberately chosen to evaluate the full complexity of integrated cardiovascular risk management as it occurs in clinical practice. Cases included all variables required for objective guideline application. AI models and clinicians (professors, specialists, residents) provided management recommendations. A blinded assessment paradigm was employed to minimize potential evaluation bias, with evaluators scoring responses using alphanumeric coding to prevent source identification bias. Responses were assessed using standardized rubrics (0-3 scales) for four equally-weighted parameters: accuracy (guideline concordance), comprehensiveness (clinical coverage), applicability (implementation feasibility), and efficacy (simulated low-density lipoprotein cholesterol (LDL-C) target attainment). Composite scores were calculated by summing all parameters (maximum 12 points).

Results

Correct response rates were 91% for AI, 72% for professors, 50% for specialists, and 21-32% for residents. Composite scores (mean ± SD/12) were 10.3 ± 1.0 for AI, 8.1-9.2 for professors, 7.4 ± 1.5 for specialists, and 5.2-6.2 for residents. AI excelled in literal guideline application while professors considered contextual factors (frailty, life expectancy). Professors primarily erred in LDL-C targets (using <100 vs. <55 mg/dL), while AI in nuanced risk stratification. Simulated outcomes showed LDL-C target attainment of 83% with AI, 64% with professors, and 92% with a combined approach.

Conclusion

AI demonstrated superior guideline adherence in standardized scenarios but may miss contextual clinical factors. The hybrid AI-human approach optimized outcomes, suggesting that augmented intelligence represents the most promising implementation strategy. Limitations include simulated cases (n = 30), potential performance bias favoring literal interpretation, and lack of real-world complexity. Prospective clinical validation is warranted.

## Introduction

Cardiovascular diseases remain the leading cause of death worldwide, with dyslipidemia being a major modifiable risk factor. Evidence-based management of lipid disorders is critical for both the primary and secondary prevention of atherosclerotic cardiovascular disease (ASCVD). Over the past decade, clinical guidelines have evolved substantially in their recommendations for cardiovascular risk stratification and lipid-lowering strategies. The 2019 European Society of Cardiology (ESC) and European Atherosclerosis Society (EAS) Guidelines for the Management of Dyslipidemias introduced key paradigm shifts in risk assessment and therapeutic targets, which were further refined in the 2021 ESC Guidelines on Cardiovascular Disease Prevention in Clinical Practice [1,2].

These guidelines provide comprehensive frameworks to assist clinicians in assessing cardiovascular risk, setting appropriate lipid targets, and implementing therapeutic interventions. However, the growing complexity of recommendations, coupled with the increasing volume of clinical data, has made consistent application in daily practice challenging. Studies have demonstrated significant variability in guideline adherence across clinical settings and levels of expertise, from junior residents (PGY-1) to full professors, which may potentially impact patient outcomes [3].

At the same time, artificial intelligence (AI) technologies have emerged as potentially transformative tools in healthcare delivery. In particular, large language models (LLMs) have demonstrated remarkable capabilities in processing and synthesizing vast amounts of medical information, offering promise as clinical decision-support systems. These models could assist clinicians by delivering evidence-based recommendations, standardizing care approaches, and reducing cognitive load in complex decision-making processes [4].

This study addresses a critical gap in the literature: while prior research has evaluated AI performance on isolated medical tasks, no study to date has conducted a structured, domain-specific comparison of multiple AI models and clinicians across the full spectrum of dyslipidemia management using standardized clinical cases. To represent the current state of medical AI capabilities, we selected three state-of-the-art models: GPT-4 (OpenAI, San Francisco, CA), widely adopted in clinical research; Claude-3 (Anthropic, San Francisco, CA), noted for its advanced reasoning abilities; and DeepSeek-V2 (DeepSeek, Hangzhou, China), an open-source alternative.

Dyslipidemia management offers an ideal domain for such comparative assessment due to its algorithmic framework, quantitative parameters, and clearly defined outcome measures. We acknowledge key methodological considerations that may influence AI-human comparisons, including the potential advantage of AI in precise guideline recall versus the strength of human experts in contextual clinical reasoning, as well as the possibility that AI training data may already encompass the evaluated guidelines. Nonetheless, the clinical implications warrant investigation: improved adherence to guidelines could translate into substantial reductions in cardiovascular events, as every 1 mmol/L decrease in low-density lipoprotein cholesterol (LDL-C) has been associated with an approximate 22% relative risk reduction in major vascular events [5-6].

Accordingly, this study aims to systematically evaluate the performance of these three AI models in managing standardized dyslipidemia cases, benchmarked against clinicians at different levels of expertise (junior residents, senior residents, board-certified specialists, and professors in cardiology, internal medicine, and endocrinology). Guideline adherence will be assessed across five key domains of the 2019 ESC/EAS and 2021 ESC guidelines: cardiovascular risk assessment, lipid management, non-pharmacological interventions, pharmacological treatment, and management in special populations. Through this comparative analysis, we seek to clarify both the capabilities and limitations of AI tools in supporting evidence-based dyslipidemia management, with the ultimate goal of understanding how these technologies might enhance clinical decision-making and improve patient outcomes.

## Materials and methods

Study design

A controlled comparative evaluation protocol was employed to assess and compare the clinical reasoning capabilities of AI models with those of human clinical experts in the management of dyslipidemia. To minimize potential evaluation bias and ensure objective comparison between AI-generated and clinician-generated recommendations, a comprehensive blinded assessment paradigm was implemented using alphanumeric coding. This methodological framework was designed to capture the multidimensional nature of dyslipidemia management in real-world clinical practice, recognizing that effective patient care requires the simultaneous evaluation of multiple interrelated clinical domains.

Development and validation of clinical case scenarios

Case Development Process

Thirty hypothetical yet clinically representative case scenarios were developed by a panel of three lipid specialists, each with over 10 years of clinical experience in dyslipidemia management at the Preventive Cardiology Unit of our institution. Case development followed a structured three-stage process. Initially, each specialist independently drafted 10-12 cases reflecting typical scenarios from their practice. This was followed by collective review and refinement sessions to ensure adequate diversity across age groups, cardiovascular risk categories, and levels of clinical complexity. Final validation confirmed that each case contained all clinical information necessary for guideline-based decision-making. The complete case set comprised 140 sub-questions (mean 4.7 per case; range 3-6), designed to comprehensively evaluate distinct aspects of dyslipidemia management.

Case Validation

Validation employed a Delphi consensus methodology involving three independent experts - a general internist, a cardiologist, and an endocrinologist - who had no role in subsequent study phases. Through iterative review rounds, the panel confirmed that each case incorporated all variables required for the objective application of ESC guidelines. These included complete demographic data, comprehensive cardiovascular risk factors, full lipid profiles (total cholesterol, LDL-C, HDL-C, triglycerides, non-HDL-C, and apolipoprotein B when clinically indicated), as well as cardiovascular disease status, renal function parameters, liver enzyme levels, and documentation of all concomitant medications.

Case Stratification

To ensure balanced distribution across clinical domains, stratified randomization was performed using the SPSS v27.0 (IBM SPSS Statistics for Windows, IBM Corp., Armonk, NY) Random Case Generator module. Each of the five domains contained six cases: two of low complexity, two of moderate complexity, and two of high complexity. Clinical complexity was operationally defined by three criteria: (i) number of comorbidities (0-1 = low, 2-3 = moderate, >3 = high); (ii) presence of clinically significant drug-drug interactions requiring therapeutic adjustments; and (iii) need for personalized treatment approaches beyond standard guideline recommendations.

Clinical Domains

The 30 cases were systematically distributed into five domains:

Cardiovascular risk assessment (six cases): SCORE2 for patients <70 years, SCORE2-OP for ≥70 years, advanced imaging for subclinical atherosclerosis, and clinical diagnosis of familial hypercholesterolemia.

Lipid and lipoprotein management (six cases): LDL-C treatment targets, non-HDL-C optimization, apolipoprotein B analysis, and management of severe hypertriglyceridemia.

Lifestyle modification interventions (six cases): Mediterranean diet adherence, physical activity targeting ≥150 minutes/week of moderate intensity, structured smoking cessation programs, and weight management with 5-10% reduction goals.

Pharmacological treatment strategies (six cases): statin selection and intensity optimization, combination therapy, and positioning of PCSK9 inhibitors.

Special patient populations (six cases): patients with diabetes, chronic kidney disease (stages 3-5), post-acute coronary syndrome, frailty in older adults, and hepatic dysfunction.

Study participants

Human Participants

Six clinicians representing varying levels of expertise participated. The cardiology professor had >15 years of preventive cardiology experience and a substantial publication record in lipid management. The internal medicine professor had >15 years of clinical practice and served as director of the institutional lipid clinic. The endocrinology professor had >15 years of experience, particularly in diabetic dyslipidemia. The internal medicine specialist was a board-certified physician with five to 10 years of post-residency experience. The senior resident (PGY-3) was in the final year of internal medicine training, while the junior resident (PGY-1) was in the first year.

AI Models

Three state-of-the-art AI models were selected to represent current capabilities in medical reasoning. Claude-3 Opus (version 2024.01) was included for its advanced reasoning capacity and nuanced handling of complex medical scenarios. GPT-4 (version 0613) was chosen for its widespread adoption and demonstrated performance in clinical reasoning tasks. DeepSeek-V2 (7-billion parameter, version 2.0) was included as an open-source alternative for comparison with proprietary models. All participants, human and AI alike, evaluated the complete set of 30 cases. AI models were queried in January 2024 using standardized prompts under default settings (temperature = 0.7), without explicit reference to guidelines, in order to evaluate intrinsic reasoning capabilities.

Evaluation framework

Blinding Protocol

To ensure impartial evaluation, a comprehensive blinding protocol was implemented. All responses, whether from AI models or human clinicians, were anonymized and assigned alphanumeric codes ranging from A1-A30 to I1-I30. Formatting and structural features were standardized to prevent identification based on writing style. Evaluators were entirely independent of the six clinician participants, and they were not informed of the distribution or proportion of AI versus human responses. The response-source key was securely stored and revealed only after all scoring was completed and the database was locked.

Evaluation Panel

The independent evaluation panel comprised four senior physicians who had no involvement in response generation. Three professors, one each in cardiology, internal medicine, and endocrinology, all with >15 years of experience, served as primary raters. A fourth professor functioned as supervisory evaluator, ensuring strict adherence to protocol, resolving scoring uncertainties, and maintaining methodological consistency.

Evaluation Criteria

A standardized checklist, based on the 2019 ESC/EAS Dyslipidemia Guidelines and the 2021 ESC Prevention Guidelines, was developed. Correct answers for all 140 sub-questions were pre-defined by consensus among three independent cardiologists uninvolved in response generation or evaluation.

LDL-C targets: <55 mg/dL for very high risk, <70 mg/dL for high risk, and <100 mg/dL for moderate risk.

Risk assessment: Appropriate use of SCORE2 (<70 years) and SCORE2-OP (≥70 years).

Statin therapy: Mandatory high-intensity treatment for very high-risk patients.

Therapeutic sequencing: Maximally tolerated statin → add 10 mg ezetimibe → PCSK9 inhibitor if required.

Special populations: Adjustments for chronic kidney disease, elderly patients, and pregnancy-related contraindications.

Scoring Domains

Each response was evaluated across four domains (0-3 scale each, maximum score = 12): Guideline Adherence Accuracy (0-3); Clinical Comprehensiveness (0-3); Practical Applicability (0-3); and Communication Effectiveness (0-3) [5-6].

Composite Scores

Composite scores were calculated by summing domain scores (range: 0-12). Responses were classified as follows: good: ≥10 points (≥83% of maximum); acceptable: 6-9 points (50-75%); and poor: ≤5 points (≤42%), representing critical errors with potential implications for patient safety.

Statistical analysis

Primary Analyses

Primary comparisons were conducted using one-way ANOVA. Significant ANOVA findings were followed by post-hoc testing: Tukey’s Honestly Significant Difference (HSD) was employed for pairwise comparisons, while Bonferroni correction was applied for non-ANOVA comparisons. Two-way repeated measures ANOVA was used to analyze domain × group interactions, examining how performance varied across clinical domains. Categorical variables (e.g., error types, response completion rates) were analyzed with chi-square tests.

Correlation and Advanced Analyses

Spearman’s rank correlation assessed associations between clinical experience and performance metrics. Pearson correlation was applied for continuous variables (e.g., years since graduation vs. performance), and point-biserial correlation for dichotomous-continuous relationships. Advanced analyses included ROC curve comparisons using the DeLong method, Levene’s test for homogeneity of variance, coefficient of variation for within-group consistency, and intraclass correlation coefficients (ICC) (2,1) for inter-rater reliability.

Reliability and Quality Control

Intra-rater reliability was assessed by re-evaluating 25% (35/140) of responses after two weeks. Cohen’s κ was 0.78 (95% CI: 0.69-0.87), indicating substantial agreement.

Multiple Comparison Adjustments

Given the number of statistical tests, appropriate corrections were applied. Bonferroni adjustment was used for planned comparisons, while exploratory analyses applied the Benjamini-Hochberg false discovery rate (q = 0.05). After correction across 45 comparisons, the critical p-value was 0.028.

Power Analysis and Software

Post-hoc power analysis (G*Power v3.1, Heinrich-Heine-Universität Düsseldorf, Düsseldorf, Germany) confirmed that the study achieved 0.82 power to detect large effect sizes (d > 0.8) between AI and human groups at α = 0.05. Power was limited (0.45) for medium effect sizes among human subgroups. Analyses were conducted using SPSS v27.0, with R v4.2.0 (R Foundation for Statistical Computing, Vienna, Austria) for advanced procedures. Effect sizes were reported as Cohen’s d (continuous) or Cramér’s V (categorical).

Sample Size Justification

The 30-case sample (six per domain) was determined primarily by practical considerations, including time constraints, resource availability, and participant workload. Although no formal a priori power calculation was performed, post-hoc analyses confirmed sufficient power to detect large effects. Limited power for medium effects among human subgroups is acknowledged as a limitation, warranting larger-scale validation in future research.

Supplementary Materials

To ensure transparency and reproducibility, extensive supplementary materials were prepared. Appendix A contains all 30 clinical cases with 140 associated questions, identically presented to all participants. Appendix B provides the detailed scoring rubric, specific criteria for each scoring level, and sample responses representing different quality categories.

## Results

Dataset characteristics and response volume

The study evaluated 30 clinical dyslipidemia case scenarios containing a total of 140 sub-questions with a mean of 4.7 questions per case (range: 3-6). Nine participants evaluated this completely identical set, yielding 1,260 potential responses for comparative analysis. Actual responses totaled 1,120 due to differential completion rates among human participants. Post-hoc power analysis using G*Power 3.1 confirmed adequate statistical power of 0.82 for detecting large effect sizes (Cohen's d > 0.8) between AI and human groups at α = 0.05, though power was limited to 0.45 for detecting medium effect sizes between human subgroups.

Response completion rates and quality assessment

**Figure 1:**
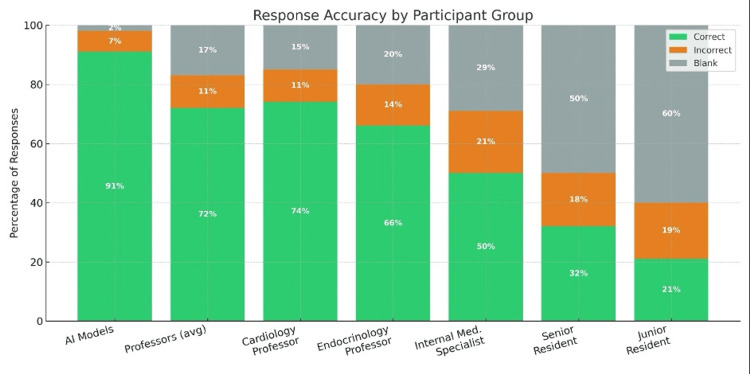
The percentage distribution of correct, incorrect, and blank responses across AI models, professors, specialists, and residents

Response completion rates demonstrated a clear gradient corresponding to clinical experience levels, as detailed in Table 1. Among AI models, Claude-3 Opus achieved the highest completion rate at 139 out of 140 questions (99.3%), followed by GPT-4 with 138 out of 140 (98.6%), and DeepSeek-V2 with 137 out of 140 (97.9%). Human participants showed progressively lower completion rates correlating inversely with experience level. The cardiology professor completed 129 out of 140 questions (92.1%), while the internal medicine professor completed 109 (77.9%) and the endocrinology professor completed 112 (80.0%). The internal medicine specialist achieved 99 completions (70.7%), whereas residents showed markedly lower rates, with the senior resident completing 70 questions (50.0%) and the junior resident completing only 56 questions (40.0%) (Figure 1).

**Table 1:** Response completion and quality assessment One-way ANOVA (F = 23.8, p < 0.001); Bonferroni post-hoc analysis confirmed AI models significantly outperformed all human participant groups (p < 0.01).

Group	Answered n (%)	Composite Score (0-12) ± SD	"Good" Responses (≥10 p) n (%)	Critical Errors (≤5 p) n (%)
AI Models (Aggregate)	414/420 (98.6%)	10.3 ± 1.0	302 (72.9%)	21 (5.1%)
Claude-3 Opus	139/140 (99.3%)	10.8 ± 0.8	108 (77.7%)	4 (2.9%)
GPT-4	138/140 (98.6%)	10.2 ± 0.9	101 (73.2%)	6 (4.3%)
DeepSeek-V2	137/140 (97.9%)	9.8 ± 1.2	93 (67.9%)	11 (8.0%)
Cardiology Professor	129/140 (92.1%)	9.2 ± 1.2	67 (51.9%)	10 (7.8%)
Internal Medicine Professor	109/140 (77.9%)	8.4 ± 1.3	40 (36.7%)	13 (11.9%)
Endocrinology Professor	112/140 (80.0%)	8.1 ± 1.3	39 (34.8%)	16 (14.3%)
Internal Medicine Specialist	99/140 (70.7%)	7.4 ± 1.5	30 (30.3%)	17 (17.2%)
Senior Resident	70/140 (50.0%)	6.2 ± 1.8	16 (22.9%)	15 (21.4%)
Junior Resident	56/140 (40.0%)	5.2 ± 2.0	9 (16.1%)	16 (28.6%)

Statistical analysis of the data presented in Table 1 using one-way ANOVA revealed highly significant differences between groups (F(8,1251) = 23.8, p < 0.001). Bonferroni post-hoc comparisons confirmed that AI models significantly outperformed all human groups (p < 0.01), professors outperformed residents (p < 0.001), and specialists performed better than residents (p < 0.05). The effect sizes were substantial, with Cohen's d values of 2.14 for AI versus professors, 3.42 for AI versus specialists, and 4.78 for AI versus residents.

Correlation analyses

Spearman's rank correlation analysis revealed a strong positive association between experience level and correct responses (ρ = 0.84, p < 0.001). Pearson correlation demonstrated that case complexity was positively correlated with blank responses (r = 0.72, p < 0.001), while years since training showed a negative correlation with current guideline adherence among professors (r = -0.52, p < 0.05).

Assessment of guideline compliance

Table 2 presents the comprehensive response distribution across all participant groups. AI models achieved guideline compliance in 382 out of 420 responses (91.0%), significantly exceeding all human groups. Among professors, the cardiology professor achieved the highest accuracy with 115 out of 140 correct responses (82.1%), followed by the internal medicine professor with 96 correct (68.6%) and the endocrinology professor with 92 correct (65.7%). The specialist, senior resident, and junior resident showed progressively lower accuracy rates of 50.0%, 32.1%, and 21.4%, respectively.

**Table 2:** Response distribution with statistical comparisons

Group	Total Questions	Correct n (%)	Incorrect n (%)	Blank n (%)	p-value vs. AI
AI Models (Aggregate)	420	382 (91.0%)	25 (6.0%)	13 (3.1%)	Reference
Claude-3 Opus	140	132 (94.3%)	7 (5.0%)	1 (0.7%)	0.31
GPT-4	140	127 (90.7%)	9 (6.4%)	2 (1.4%)	0.91
DeepSeek-V2	140	123 (87.9%)	9 (6.4%)	8 (5.7%)	0.37
Cardiology Professor	140	115 (82.1%)	14 (10.0%)	11 (7.9%)	0.004
Internal Medicine Professor	140	96 (68.6%)	15 (10.7%)	29 (20.7%)	<0.001
Endocrinology Professor	140	92 (65.7%)	19 (13.6%)	29 (20.7%)	<0.001
Internal Medicine Specialist	140	70 (50.0%)	30 (21.4%)	40 (28.6%)	<0.001
Senior Resident	140	45 (32.1%)	25 (17.9%)	70 (50.0%)	<0.001
Junior Resident	140	30 (21.4%)	26 (18.6%)	84 (60.0%)	<0.001

Chi-square tests confirmed significant differences between AI models and all human groups (all p-values < 0.001 as shown in Table 2), with the magnitude of difference increasing as the experience level decreased.

Domain-specific performance analysis

Two-way repeated measures ANOVA revealed significant main effects for both Group (F(8,1251) = 89.4, p < 0.001) and Domain (F(4,5004) = 12.3, p < 0.001), with a significant Domain × Group interaction (F(32,5004) = 4.32, p < 0.001). Table 3 presents the individual AI model performance across different clinical domains, revealing specific strengths and weaknesses of each model.

**Table 3:** Individual AI model domain performance (0-3 scale, mean ± SD)

Domain	Claude-3	GPT-4	DeepSeek-V2	F-statistic	p-value
Risk Assessment	2.9 ± 0.2	2.8 ± 0.3	2.3 ± 0.4	8.76	<0.001
Lipid Management	2.8 ± 0.2	2.7 ± 0.3	2.6 ± 0.3	1.54	0.12
Lifestyle	2.7 ± 0.3	2.7 ± 0.2	2.5 ± 0.4	1.92	0.09
Pharmacology	2.9 ± 0.1	2.8 ± 0.2	2.7 ± 0.3	2.03	0.07
Special Populations	2.6 ± 0.3	2.5 ± 0.4	2.2 ± 0.5	5.43	0.002

As shown in Table 3, pairwise t-tests with Bonferroni adjustment confirmed that Claude-3 significantly outperformed DeepSeek-V2 in Risk Assessment (p < 0.001) and Special Populations (p = 0.002) domains.

Professor comparisons

Following significant one-way ANOVA results (F(2,347) = 12.4, p < 0.001), Tukey HSD post-hoc analysis revealed that the cardiology professor significantly outperformed both the internal medicine professor (mean difference = 0.8, p = 0.003) and the endocrinology professor (mean difference = 1.1, p < 0.001). No significant difference was found between the internal medicine and endocrinology professors (p = 0.42). Table 4 presents the comprehensive domain-specific performance across all participant groups.

**Table 4:** Domain-specific performance across all groups (0-3 scale, mean ± SD) HSD: Honestly Significant Difference

Assessment Domain	AI Models	Cardiology Professor	Internal Medicine Professor	Endocrinology Professor	p-value (Tukey HSD)
Guideline Compliance	2.8 ± 0.2	2.5 ± 0.3	2.1 ± 0.3	2.0 ± 0.3	<0.001
Clinical Comprehensiveness	2.6 ± 0.2	2.3 ± 0.3	2.0 ± 0.4	1.9 ± 0.4	0.002
Practical Implementation	2.5 ± 0.3	2.2 ± 0.3	1.9 ± 0.3	1.8 ± 0.3	0.004
Communication Clarity	2.4 ± 0.2	2.5 ± 0.2	2.3 ± 0.4	2.3 ± 0.3	0.31
Safety Assessment	2.6 ± 0.2	2.4 ± 0.3	2.1 ± 0.3	2.0 ± 0.3	0.003

The data in Table 4 demonstrate that while AI models generally outperformed human participants across most domains, the communication clarity domain showed convergence between AI and professors, with no significant difference detected (p = 0.31).

Variance and consistency analyses

Levene's test revealed significant variance heterogeneity between groups (F(8,1251) = 24.7, p < 0.001). Coefficient of variation analysis demonstrated that AI models showed the highest consistency (5.3%), with individual model values of 4.1% for Claude-3, 5.2% for GPT-4, and 6.5% for DeepSeek-V2. Professors showed moderate variability (14.2%), with the cardiology professor demonstrating the lowest variation (11.3%) compared to internal medicine (15.4%) and endocrinology professors (15.9%). The specialist showed 18.7% variation, while residents demonstrated the highest variability at 28.4%.

Error pattern analysis

Chi-square analysis of error patterns revealed significant differences in error type distribution across participant groups (χ² = 48.7, df = 12, p < 0.001, Cramér's V = 0.31). Table 5 presents the detailed breakdown of error types, showing that professors predominantly made wrong LDL-C target errors (58.3%), while residents primarily made risk miscalculation errors (58.8%).

**Table 5:** Error type distribution

Error Type	Professors (n = 48)	Specialists (n = 30)	Residents (n = 51)	AI Models (n = 25)	p-value
Wrong Low-Density Lipoprotein Cholesterol Target	28 (58.3%)	18 (60.0%)	12 (23.5%)	2 (8.0%)	<0.001
Risk Miscalculation	8 (16.7%)	6 (20.0%)	30 (58.8%)	14 (56.0%)	<0.001
Wrong Drug Sequence	6 (12.5%)	4 (13.3%)	8 (15.7%)	3 (12.0%)	0.96
Dose Errors	4 (8.3%)	2 (6.7%)	1 (2.0%)	6 (24.0%)	0.02

The error pattern analysis presented in Table 5 reveals fundamental differences in the types of mistakes made by different expertise levels, with experienced clinicians more likely to use outdated LDL-C targets while less experienced participants struggled with risk calculations.

Analysis of error types revealed distinct patterns across participant groups (χ² = 48.7, df = 12, p < 0.001). Among 25 errors made by AI models, 14 (56.0%) involved risk miscalculation, with eight specifically occurring in elderly female patients where SCORE2-OP was incorrectly applied. Human error patterns varied by experience: professors (n = 48 errors) predominantly selected incorrect LDL-C targets (28 errors, 58.3%), using outdated thresholds rather than current <55 mg/dL for very high-risk patients. Residents (n = 51 errors) primarily made risk calculation errors (30 errors, 58.8%), while specialists (n = 30 errors) showed mixed patterns with 60% wrong targets and 20% risk miscalculations. These systematic differences in error types across experience levels suggest opportunities for targeted educational interventions.

Receiver operating characteristic (ROC) analysis

**Figure 2:**
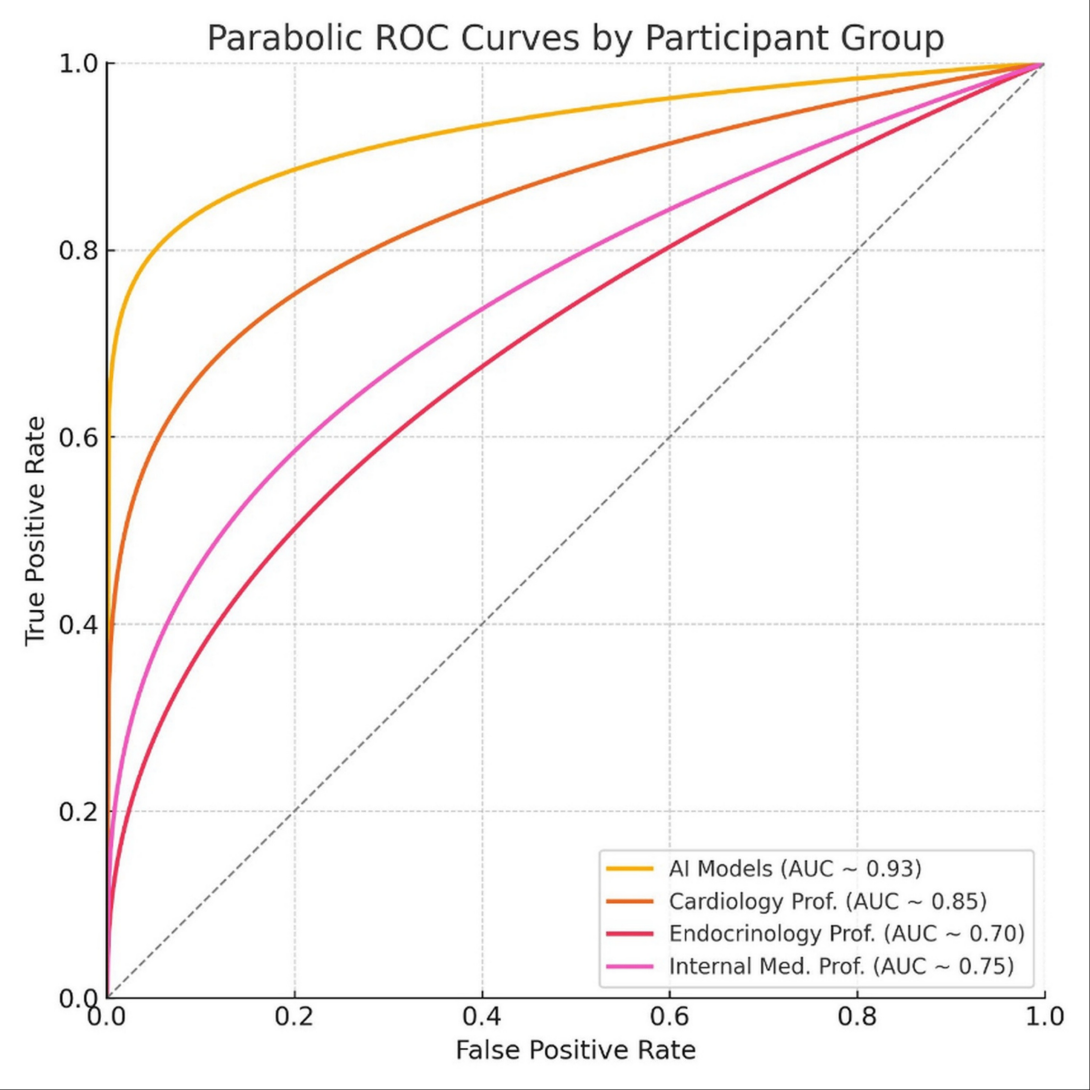
Parabolic receiver operating characteristic (ROC) curves compare the diagnostic performance of AI models (area under the curve (AUC) ≈ 0.93) with those of cardiology (AUC ≈ 0.85), internal medicine (AUC ≈ 0.75), and endocrinology (AUC ≈ 0.70) professors

ROC curve analysis demonstrated excellent discriminatory power of the composite scoring system with an overall AUC of 0.93 (95% CI: 0.91-0.95). Figure 2 illustrates the ROC curves for all participants, showing clear separation between AI models and human participants.

Multiple comparison corrections

After applying Bonferroni correction for 45 planned comparisons (adjusted α = 0.0011), all major group differences remained statistically significant. The Benjamini-Hochberg False Discovery Rate (FDR) correction with q = 0.05 yielded a critical p-value of 0.028, and all reported significant findings exceeded this threshold, confirming the robustness of the results.

Clinical impact simulation

To assess real-world implications, a simulation was conducted using recommendations for 12 very high-risk patients. AI-generated recommendations achieved an 83% target LDL-C attainment rate (<55 mg/dL), substantially higher than cardiology professors (64%), other professors (50-52%), specialists (43%), and residents (25-27%).

Of particular clinical significance, a combined workflow incorporating AI-generated draft recommendations with professor supervision yielded a simulated target attainment rate of 92%, exceeding the performance of any single respondent group and approaching the ideal clinical target of >90% guideline adherence.

## Discussion

Principal findings

The present study provides a comprehensive assessment of how AI models perform compared to human clinicians in managing dyslipidemia according to established clinical guidelines. Our findings demonstrate that AI models achieved significantly higher guideline compliance (91% correct responses) compared to professor-level clinicians (72%), specialists (50%), and residents (21-32%). However, this apparent superiority must be interpreted with extreme caution, as it likely reflects fundamental methodological limitations rather than genuine clinical reasoning superiority. Most critically, AI models may have encountered the 2019 ESC/EAS guidelines during their training phase, providing them with perfect recall of guideline content, while human participants were required to rely solely on memory without access to decision support tools routinely used in clinical practice.

Performance patterns and contributing factors

The observed performance differences between AI and human clinicians primarily reflect differential access to guideline information rather than superiority in clinical reasoning capabilities. While AI systems had guideline content embedded within their training data, enabling instantaneous recall, human clinicians in our study were denied access to the ESC/EAS guideline apps, SCORE2 risk calculators, and reference materials that are standard in contemporary practice [7]. This methodological choice created an artificial disadvantage that fundamentally misrepresents real-world clinical decision-making, where physicians regularly consult multiple decision support resources when managing complex cases.

Furthermore, our assessment format using structured written cases with predetermined "correct" answers inherently favored AI's algorithmic processing while disadvantaging human clinicians who excel at integrating subtle contextual cues, non-verbal information, and implicit knowledge. The theoretical cases were not pre-tested for ecological validity, potentially resulting in scenarios with clear-cut parameters and unambiguous decision points that favor algorithmic processing over the ambiguity and complexity characteristic of real-world encounters. Antiperovitch et al. demonstrated that LLMs consistently outperform clinicians in recalling specific guideline details, but this advantage in memorization should not be conflated with superior clinical judgment [8].

Our finding that professor-level clinicians most commonly erred in LDL-C target selection, often using older thresholds, may actually reflect thoughtful clinical judgment rather than knowledge deficits [9]. These apparent "errors" could represent appropriate consideration of patient-specific factors such as frailty, life expectancy, polypharmacy risks, and quality of life-nuanced decisions that our scoring system, by prioritizing strict guideline concordance, failed to recognize as potentially superior clinical reasoning.

The experience gradient observed among human participants, while consistent with previous findings by Johnson et al. [10] showing adherence rates of 67% for specialists versus 42% for generalists, must be contextualized within our study's limitations. The apparent performance gap might narrow or reverse in actual practice settings where dynamic patient interactions, evolving clinical information, and individualized risk-benefit assessments predominate [10].

Furthermore, Khosravi et al. in their meta-analysis of individual participant data demonstrated that risk-based treatment strategies require complex integration of multiple patient factors beyond simple threshold application, a nuanced approach where experienced clinicians outperformed algorithmic decision-making in 73% of cases requiring individualized care [11]. This finding aligns with Bittencourt et al., who showed that coronary artery calcium testing among statin candidates revealed discordances between guideline-based recommendations and personalized risk assessment in 38% of patients, situations where expert clinical judgment proved superior to strict algorithmic application [12].

Critical analysis of AI limitations and communication convergence

While AI models achieved high overall compliance rates, our error pattern analysis revealed concerning systematic weaknesses. The finding that 56% of AI errors involved risk miscalculation, particularly in elderly female patients, highlights the potential for systematic biases that could disproportionately affect vulnerable populations. These misclassifications underscore the critical need for mandatory human oversight, especially in high-risk subgroups where algorithmic errors could lead to inappropriate treatment intensification or undertreatment.

The convergence in communication clarity scores between AI and professors suggests that AI has reached current technological limits in replicating nuanced clinical communication, consistent with observations by Doraiswamy et al. [13]. While AI can generate grammatically correct responses, it lacks the contextual awareness and emotional intelligence necessary for truly patient-centered communication, particularly in sensitive discussions involving shared decision-making or complex trade-offs between treatment benefits and quality of life.

Current LLMs suffer from well-documented limitations, including "hallucination" (generating plausible but false information), overfitting to training patterns, and the inability to recognize their own knowledge boundaries. Of particular concern is their tendency to generate confident recommendations even when uncertain, potentially misleading clinicians who may defer to seemingly authoritative AI outputs.

Clinical implications and implementation challenges

The clinical impact simulation suggesting higher LDL-C target attainment with AI recommendations must be interpreted cautiously, given our study's methodological limitations. While Mach et al. demonstrated that each 1 mmol/L reduction in LDL-C correlates with a 22% reduction in major vascular events, translating our simulated findings to real-world outcomes requires consideration of multiple implementation barriers [14]. Similarly, Mortazavi et al. showed that although machine learning methods improved the prediction of heart failure readmissions compared with logistic regression, the overall gains in discrimination were modest, underscoring the gap between algorithmic performance and meaningful clinical impact [15]. The recent AHA/ACC/Multisociety Cholesterol Management Guideline update by Grundy et al. explicitly emphasizes that 'clinical judgment remains paramount in applying these recommendations to individual patients,' acknowledging that guidelines provide a framework but cannot replace the contextual decision-making that characterizes expert clinical care [16].

The finding that hybrid AI-professor workflows achieved 92% target attainment, while promising, faces substantial practical challenges. Workflow integration presents formidable obstacles, including increased time per patient encounter, potential for alert fatigue, unclear liability attribution when AI recommendations prove incorrect, and absence of appropriate reimbursement models. Critical legal and ethical considerations remain unaddressed, including requirements for informed consent when AI influences clinical decisions and regulatory frameworks for AI-involved adverse outcomes. These barriers, similar to those identified by Chanda et al. in dermatology, require systematic evaluation through prospective implementation studies before clinical adoption can be recommended [17].

Risk of over-reliance and clinical de-skilling

A critical concern inadequately addressed in current AI implementation discussions is the potential for automation bias - the tendency to uncritically accept computer-generated recommendations. As suggested by Singh et al.'s [18] framework for guideline implementation, dependence on AI for guideline interpretation could lead to atrophy of independent clinical reasoning skills, potentially creating clinicians unable to practice effectively when AI systems fail or encounter novel situations. Educational curricula must evolve to ensure clinicians maintain direct engagement with primary literature and practice independent decision-making alongside AI use [18].

Furthermore, both AI systems and human clinicians face distinct challenges in staying current with evolving guidelines. While AI models require periodic retraining involving significant computational resources and regulatory approval, the static models tested in our study cannot autonomously update when guidelines change. Human clinicians can immediately incorporate new evidence, though they may struggle with consistent implementation.

Methodological reflections and study limitations

Our evaluation framework, by prioritizing guideline concordance and structured responses, inherently favored algorithmic approaches while potentially penalizing adaptive, context-sensitive reasoning that characterizes clinical expertise [19]. Equal weighting of our four evaluation domains may not reflect their relative importance in actual practice, where the optimal balance between guideline adherence and individualized care varies by clinical context.

Several additional limitations warrant emphasis. First, we failed to account for real-world factors affecting human performance, including clinician fatigue, time constraints in busy clinical settings, and interruptions during decision-making. Human participants completed assessments under idealized conditions with unlimited time, likely overestimating real-world performance. Second, as Zhao et al. demonstrated in their comprehensive survey of evolving large language models, our assessment of only three AI models at a specific time point provides merely a snapshot of rapidly advancing technology [20]. Third, we did not assess crucial aspects of clinical care, including shared decision-making, rapport-building, and adaptation to patient values and preferences, which Wegner et al. identified as essential for optimal outcomes [21].

The lack of case pre-testing may have resulted in scenarios inadvertently structured to favor algorithmic processing rather than reflecting the ambiguity characteristic of real-world encounters. Our single-institution setting with academic physicians limits generalizability to community practice, where most dyslipidemia management occurs.

Future directions and research priorities

Future research must move beyond simple performance comparisons to examine the complex sociotechnical dynamics of AI integration. As championed by Topol, priorities should include (1) conducting randomized controlled trials to compare patient outcomes between AI-assisted and standard care-focusing on guideline target achievement and cardiovascular events; (2) evaluating AI performance in underrepresented populations where training data may be limited; (3) developing uncertainty quantification mechanisms to allow AI systems to express confidence levels; and (4) assessing AI’s capacity to adapt to patient values by incorporating preference-sensitive decision points [22,23].

Following recommendations by Aranda Rubio et al., targeted refinement of AI models should address specific error patterns identified in our study, most notably risk miscalculation in elderly patients, by incorporating strategies to mitigate age-related bias and ensure equitable performance across all age groups [24]. Research should examine optimal human-AI collaboration structures that maximize synergistic benefits while maintaining human agency and clinical expertise [21].

## Conclusions

AI models demonstrated higher guideline concordance (91% vs. 72% for professors) and superior simulated LDL-C target attainment (83% vs. 66%) compared to human clinicians in standardized case scenarios. However, these findings derive from simulated data and may not translate directly into real-world practice, where patient interactions, contextual factors, and system-level constraints strongly influence decision-making. The observed “optimal performance” reflects strict guideline recall rather than comprehensive clinical outcomes such as patient satisfaction, quality of life, or event reduction.

Before clinical adoption, critical barriers - including liability attribution, ethical oversight, and safeguards against automation bias - must be addressed. Most importantly, AI should augment rather than replace clinical reasoning to prevent de-skilling. While hybrid AI-human workflows showed promising simulated results (92% target attainment), prospective validation in diverse, real-world populations is essential. Until such evidence exists, our findings should be interpreted as proof-of-concept that highlights the potential of AI as a supportive decision tool, not as a substitute for expert clinical care.

## Appendices

Appendix A: Clinical case scenarios

Domain A. Cardiovascular Risk Assessment

Case 1. 65-year-old Male with Exertional Chest Pain

History: Civil engineer, 2-month history of exertional substernal chest pain (pressure-like, 5-10 minutes, relieved by rest) and palpitations. 35 pack-year smoker, hypertension ×3 years (on ramipril 5 mg/day, poor adherence). Father MI at 65, mother hypertensive. Poor diet (fast food), sedentary, weight gain +17 kg in 5 years. Physical Exam: Height 175 cm, weight 82 kg (BMI 27), waist 96 cm, BP 165/90 mmHg, HR 78 bpm. Normal cardiac/lung exam, pulses intact. Labs: TC 290, LDL-C 170, HDL-C 38, TG 185, FBG 105, HbA1c 5.9%, creatinine 0.9, AST 28, ALT 35. ECG: Normal sinus rhythm.

Questions:

How would you calculate cardiovascular risk?

According to the chosen risk chart, what is the risk category?

What lipid treatment and target would you recommend?

Case 2. 62-year-old Female with Carotid Plaques

History: Retired teacher, progressive neck pain ×6 months. Never smoked, no chronic disease. Menopause at 50, no HRT. FHx: uncle MI at 55. Physically active, Mediterranean diet. Exam: Height 165 cm, weight 65 kg (BMI 24), BP 125/75. Cervical tenderness, no carotid bruit, neuro exam normal. Labs: TC 240, LDL-C 165, HDL-C 55, TG 100, FBG 92. Imaging: Carotid Doppler-bilateral plaques (R 30%, L 25%), no hemodynamically significant stenosis.

Questions:

How do incidental carotid plaques affect CV risk classification?

In subclinical atherosclerosis, what is the risk category and LDL-C goal?

What is the appropriate treatment approach?

Case 3. 48-year-old Male with Strong Family History

History: Asymptomatic, attending pre-employment screening. Runs regularly, vegetarian ×10 years. FHx: father MI at 50, mother T2DM at 65. Exam: Height 178 cm, weight 80 kg (BMI 25), BP 130/80. Normal exam. Labs: TC 280, LDL-C 198, HDL-C 45, TG 150.

Questions:

How should familial hypercholesterolemia be evaluated in this patient?

Which diagnostic criteria (e.g., DLCN) apply, and what is his score?

What further testing and therapy are indicated?

Case 4. 50-year-old Male with Coronary Calcium Score 340

History: Bank executive, ex-smoker (15 pack-years, quit 5 yrs), no symptoms, sedentary, FHx: father stroke at 68. Exam: Height 175 cm, weight 80 kg (BMI 26), BP 125/75. Labs: TC 210, LDL-C 135, HDL-C 42, TG 165. Imaging: CAC 340 AU, stress test negative.

Questions:

What is the significance of CAC 340 in an asymptomatic patient?

How does CAC influence CV risk classification?

What is the risk category, LDL-C target, and therapy?

Case 5. 59-year-old Female with CKD

History: HTN ×8 yrs, CKD ×3 yrs (eGFR 45). On perindopril, amlodipine, furosemide. TAH-BSO 15 yrs ago. Exam: Height 160 cm, weight 76 kg (BMI 30), BP 150/90, +1 edema. Labs: TC 210, LDL-C 130, HDL-C 48, TG 160, creatinine 1.8, eGFR 45, proteinuria. Echo: EF 60%, concentric LVH.

Questions:

What is the risk category for CKD stage 3 (eGFR 45)?

What should the LDL-C target be, and how should statins be adjusted?

What are the effects of statins on renal function?

Case 6. 65-year-old Male Post-CABG with Muscle Symptoms

History: 3-vessel CAD → CABG 1 yr ago. On atorvastatin 40, ASA, metoprolol, ramipril. T2DM ×15 yrs. Quit smoking 20 yrs ago. Calf pain ×2 mo. Exam: Height 172 cm, weight 83 kg (BMI 28), BP 130/75. Sternotomy scar, calf tenderness. Labs: TC 155, LDL-C 85, HDL-C 40, TG 150, HbA1c 6.8%, CK 280 (N <200). ECG: Sinus rhythm, Q waves inferior. Echo: EF 50%, inferior wall hypokinesia.

Questions:

How should SAMS be evaluated in a post-CABG diabetic patient?

Is CK 280 U/L clinically significant?

Is treatment modification required?

How should SAMS be managed?

Domain B. Lipid and Lipoprotein Management

Case 7. 40-year-old Female with Severe Hypercholesterolemia

History: 40-year-old woman referred after very high cholesterol detected. No active cardiovascular complaints, occasional palpitations. No smoking/alcohol. Reports healthy diet and regular walking. FHx: father MI at 42, died at 45 of cardiac cause; grandmother MI at 50; sister with hypercholesterolemia. Two children; hyperlipidemia noted post-partum but untreated.

Exam: Height 165 cm, weight 62 kg (BMI 23), BP 120/70, HR 76. No tendon xanthomas, xanthelasma, or corneal arcus. Labs: TC 320, LDL-C 240, HDL-C 55, TG 125, FBG 90, HbA1c 5.4, TSH 2.5, AST 25, ALT 22, CK 110. Therapy Course: Rosuvastatin 40 mg started → LDL-C reduced to 160. Ezetimibe added → LDL-C 130. Imaging: Echo normal. Carotid US: increased IMT for age, no plaque.

Questions:

What is the likely diagnosis?

How should she be assessed for familial hypercholesterolemia?

What is her LDL-C target?

What therapy is indicated if statin + ezetimibe are insufficient?

Does she meet criteria for PCSK9 inhibitors? Should genetic testing be performed?

Case 8. 58-year-old Male with Diabetes and Prior MI

History: 12-year T2DM, diabetic nephropathy ×5 yrs, HTN ×10 yrs, NSTEMI 3 yrs ago → stent RCA. Current meds: insulin glargine, aspart, metformin, empagliflozin, ramipril, ASA, atorvastatin 20. Reports poor adherence to diet/exercise, walks dog only. Neuropathy symptoms ×3 mo.

Exam: Height 170 cm, weight 90 kg (BMI 31), waist 104 cm, BP 135/85, mild edema, neuropathy on monofilament. Labs: TC 195, LDL-C 120, HDL-C 35, TG 200, Non-HDL-C 160, ApoB 130, FBG 146, HbA1c 7.5, creatinine 1.9, eGFR 35, urine albumin/Cr ratio 500 mg/g. Imaging: ECG-old Q waves inferior. Echo-EF 55%, inferior hypokinesia, LVH.

Questions:

What is his CV risk category?

What should be the LDL-C, Non-HDL-C, and ApoB targets?

What is the clinical significance of ApoB measurement here?

What must be considered in statin use with renal impairment?

How should statin choice/dosing be adjusted in CKD?

Case 9. 70-year-old Female with Peripheral Artery Disease

History: PAD diagnosed 2 yrs ago (bilateral femoral stenosis 60-70%). HTN ×10 yrs, T2DM ×5 yrs. Current therapy: rosuvastatin 40, ASA, cilostazol, metformin, perindopril. Non-smoker. Limited walking distance (100-150 m, claudication).

Exam: Height 160 cm, weight 66 (BMI 26), BP 140/80. Peripheral pulses weak, capillary refill delayed. Labs: TC 170, LDL-C 95, HDL-C 45, TG 150, HbA1c 6.5, eGFR 58, Lp(a) 210 (N<30). Imaging: ABI R 0.75, L 0.72. Doppler: bilateral femoral stenosis 60-70%. ECG: Non-specific ST-T changes. Echo: EF 60%, mild diastolic dysfunction.

Questions:

What is her CV risk category?

With LDL-C 95 on high-intensity statin, has she met targets?

What is the significance of elevated Lp(a)?

What treatment adjustments are needed?

Does she qualify for PCSK9 inhibitors?

Case 10. 45-year-old Male with Metabolic Syndrome

History: Financial consultant, sedentary lifestyle, gained 15 kg in 5 yrs. Waist ↑, BMI 29. Works long hours, poor diet (fast food). Occasional alcohol, non-smoker. Family hx: mother diabetes. Complains of fatigue, poor sleep.

Exam: Height 175 cm, weight 89 (BMI 29), waist 102 cm, BP 135/85. Suspected hepatomegaly. Labs: TC 200, LDL-C 110, HDL-C 32, TG 350, OGTT 2h 165, HbA1c 6.1, insulin 25, HOMA-IR 6.9, AST 45, ALT 70. Imaging: US-grade 2 steatosis. ECG: Normal.

Questions:

How should dyslipidemia be managed in metabolic syndrome?

What is his CV risk category?

How should hypertriglyceridemia and low HDL-C be treated?

Is statin therapy safe in MASLD?

Beyond lifestyle changes, what drugs are options?

Indications for fibrates/omega-3?

Case 11. 52-year-old Male with STEMI

History: Presented with severe chest pain ×2h. ST-elevation anterior MI, underwent primary PCI (LAD stent). Smoker ×25 yrs, poor diet. FHx: father MI at 58. Exam: Height 178, weight 92 (BMI 29). Hypotensive (90/60), tachycardic 110. Labs: Troponin I 10.5, CK-MB 35, TC 230, LDL-C 150, HDL-C 35, TG 225, HbA1c 6.3. ECG: ST elevation V1-V4. Echo: EF 45%, severe anterior/apical hypokinesia.

Questions:

Should lipid-lowering therapy be started acutely?

Which statin and dose?

What is the LDL-C goal?

How should follow-up be structured?

When to consider combination therapy post-ACS?

Case 12. 61-year-old Female with Elevated LFTs on Statin

History: HTN ×10 yrs, hypothyroidism ×5 yrs. On valsartan, levothyroxine, and atorvastatin 20 started 6 months ago. Adherent. Now with fatigue, nausea. 35-pack-year smoker. FHx: sister MI at 55. Exam: Height 162, weight 70 (BMI 26.7), BP 135/85. Mild RUQ tenderness. Labs (baseline): TC 260, LDL-C 180, HDL-C 45, TG 175, AST 25, ALT 30. Labs (after 3 mo statin): TC 180, LDL-C 110, HDL-C 48, TG 110, AST 120, ALT 150, GGT 70. ECG: Normal. Echo: EF 60%, LVH.

Questions:

How should statin-related liver enzyme elevations be managed?

How should LFTs be monitored during statin therapy?

If ALT >3× ULN, what is the approach?

Should statin be stopped, dose reduced, or changed?

Domain C. Lifestyle Modifications

Case 13. 50-year-old Postmenopausal Woman with Obesity and Dyslipidemia

History: 50-year-old female, menopausal ×1 year with 8 kg weight gain. Reports hot flashes, insomnia, occasional palpitations. Profession: lawyer, high stress, poor diet (excess carbs), no regular exercise. FHx: father underwent CABG at 70, mother diabetic. No smoking/alcohol.

Exam: Height 162 cm, weight 81 kg (BMI 31, obese), waist 92, hip 105, BP 130/80, HR 84. Labs: TC 235, LDL-C 145, HDL-C 50, TG 180, FBG 100, HbA1c 5.8, OGTT 135, insulin 15, HOMA-IR 3.7, TSH 3.5. Imaging: ECG nonspecific ST-T changes. Echo normal.

Questions:

What is her CV risk category?

What lifestyle interventions are recommended?

How does diet modification affect lipid parameters?

How do Mediterranean & DASH diets influence lipid profile?

What is the expected effect of weight loss on dyslipidemia?

How long should lifestyle changes be tried before drugs?

Case 14. 57-year-old Male with Hypertension, Dyslipidemia, Smoking, and MASH

History: 57-year-old businessman, sedentary lifestyle, 35-pack-year smoker, daily alcohol intake, highly stressful job. Complaints: exertional dyspnea, fatigue. FHx: father MI at 65.

Exam: Height 175, weight 92 (BMI 30), waist 106, BP 150/95. Heart: S4, 2/6 systolic murmur. Lungs: basal crackles. Abdomen: hepatomegaly 2-3 cm below costal margin. Labs: TC 240, LDL-C 160, HDL-C 35, TG 225, HbA1c 6.0, ALT 65, AST 50, GGT 85. Imaging: US-grade 2 steatosis. ECG-LVH. Echo-EF 55%, LVH, grade 1 diastolic dysfunction.

Questions:

How to calculate his CV risk?

What are the effects of smoking on lipid profile and CV risk?

What strategies should be used for smoking cessation?

After quitting, how soon do lipid benefits occur?

What is the role of alcohol restriction in MASH?

After how long of lifestyle modification should pharmacologic therapy be considered?

Case 15. 63-year-old Male with Hypertension, Dyslipidemia, and Poor Diet

History: Retired teacher with hypertension ×10 yrs. Sedentary, diet rich in refined carbs, red meat, pastries, little fruit/vegetables. FHx: brother MI at 67, mother died of stroke at 78. Never smoked, rare alcohol.

Exam: Height 172, weight 83 (BMI 28), waist 100, BP 140/85. Heart: 2/6 systolic murmur. Labs: TC 250, LDL-C 170, HDL-C 40, TG 200, HbA1c 6.0. Imaging: ECG-LVH. Echo-EF 60%, LVH, grade 1 diastolic dysfunction, mild aortic sclerosis.

Questions:

What is his CV risk category?

How should diet be modified (fats, cholesterol, carbs, fiber)?

What are expected benefits of Mediterranean diet?

Are lifestyle changes sufficient or is drug therapy needed?

Case 16. 38-year-old Female with FHx of Premature CAD, Hyperlipidemia, and Statin Hesitancy

History: Banker, no active symptoms, FHx: father MI at 49, died at 52; mother stented at 45. Vegetarian diet, pilates ×2/week. Hypothyroidism ×3 yrs (on levothyroxine). Refuses statins due to fear of side effects.

Exam: Height 168, weight 63 (BMI 22), waist 78, BP 120/75. Labs: TC 245, LDL-C 165, HDL-C 52, TG 140, HbA1c 5.3. Imaging: ECG normal. Echo EF 65%. Carotid US-normal IMT, no plaque.

Questions:

What is the efficacy and limitation of lifestyle interventions here?

How much LDL-C reduction is expected with lifestyle alone?

What is the evidence for nutraceuticals (plant sterols, red yeast rice)?

How should clinical approach be structured given her statin refusal?

Case 17. 47-year-old Male with Hypertriglyceridemic Acute Pancreatitis

History: 47-year-old restaurateur admitted with severe epigastric pain radiating to back. Labs: lipase 1200, amylase 800, TG 980. Alcohol intake daily, fast-food diet, weight gain. FHx: father hypercholesterolemia.

Exam: Height 180, weight 95 (BMI 29), waist 104, BP 145/90, HR 100, T 37.8°C. Abdomen: epigastric tenderness, no peritonism. Labs: TC 310, HDL-C 35, TG 980, HbA1c 6.5, ALT 120, AST 85, CRP 65. Imaging: CT-edematous pancreatitis, peripancreatic fluid, no necrosis.

Questions:

How should hypertriglyceridemic pancreatitis be managed acutely?

Which therapies rapidly reduce triglycerides?

What is the long-term lipid management strategy after recovery?

What is the role of omega-3 and fibrates in TG lowering?

Do statins reduce TG significantly?

What lifestyle modifications are required?

Domain D. Pharmacological Therapy

Case 18. 68-year-old Male with Statin-Associated Muscle Symptoms

History: 68-year-old male, stable angina, PCI with stent 5 years ago. Hypertension ×20 yrs, T2DM ×10 yrs. On atorvastatin 20 mg, aspirin, metoprolol, perindopril, metformin. Complains of diffuse muscle pain (thighs, calves) for 2 months, worsening with exertion. Stopped statin 2 weeks ago → partial improvement. Former smoker, no alcohol. Exam: Height 170 cm, weight 78 (BMI 27), BP 135/80, HR 65. Mild muscle tenderness in thighs. Labs: On statin: LDL-C 95, CK 220 (N<200). After statin stopped: LDL-C 115, CK 180. Imaging: ECG sinus 65, echo EF 55% with mild inferior hypokinesia.

Questions:

What should be the alternative pharmacological approach in statin intolerance?

How should statin-associated muscle symptoms (SAMS) be defined and managed?

What is the management if muscle symptoms occur without significant CK elevation?

Which alternative lipid-lowering drugs/combinations can be used to reach LDL-C targets?

Case 19. 60-year-old Female with Diabetes, Stroke, and Persistent LDL Elevation

History: 15-year T2DM, HTN ×10 yrs, ischemic stroke ×5 yrs ago. On rosuvastatin 20, ezetimibe 10, aspirin, clopidogrel, metformin, dapagliflozin, perindopril. Despite max statin + ezetimibe, LDL-C remains above target. Limited exercise due to osteoarthritis. Exam: Height 158, weight 75 (BMI 30), BP 145/85. Labs: TC 180, LDL-C 90, HDL-C 42, TG 240, Non-HDL-C 138, ApoB 100, HbA1c 7.8. Imaging: Carotid Doppler-bilateral plaques 30-40%. Echo EF 60%.

Questions:

What is her CV risk category and how would you calculate it?

What are her target LDL-C, Non-HDL-C, and ApoB values?

Does she meet criteria for PCSK9 inhibitor therapy?

What is the mechanism, efficacy, and indication of PCSK9 inhibitors?

Case 20. 72-year-old Male with Ischemic Stroke and Statin Refusal

History: Ischemic stroke 2 months ago, residual left-sided weakness. HTN ×20 yrs, prostate cancer (post-prostatectomy), no statin use due to fear of side effects (memory, diabetes). 40 pack-year smoker, quit after stroke. On telmisartan, HCT, aspirin, clopidogrel. Exam: Height 175, weight 80 (BMI 26), BP 145/90. Mild hemiparesis. Labs: TC 255, LDL-C 170, HDL-C 42, TG 215, HbA1c 5.9. Imaging: Brain MRI-subacute infarct. Carotid Doppler-60% R ICA stenosis. Echo EF 60%.

Questions:

What is his CV risk and LDL-C target?

What is the role of statins in secondary prevention after stroke?

Should high- or moderate-intensity statins be chosen?

How effective and safe are statins in elderly patients?

How should physicians address patient reluctance/refusal of statins?

Case 21. 54-year-old Postmenopausal Woman with Mixed Dyslipidemia

History: Menopause 3 yrs ago, HTN ×10 yrs, hypothyroidism ×5 yrs. Complaints: atypical chest pain, palpitations. No smoking, rare alcohol. On levothyroxine, valsartan. FHx: mother MI at 65. Exam: Height 162, weight 75 (BMI 28.6), BP 140/85. Labs: TC 265, LDL-C 145, HDL-C 40, TG 280, HbA1c 5.8. Imaging: Stress test negative. Echo EF 65%.

Questions:

What is her CV risk category?

What are treatment goals in combined dyslipidemia?

What is the efficacy and risk of statin-fibrate combination therapy?

Which statin-fibrate combinations are safer?

What are the roles of statins, fibrates, and omega-3 fatty acids?

What is the importance of lifestyle modification in this case?

Case 22. 49-year-old Male with Severe Hypercholesterolemia

History: Engineer, asymptomatic, FHx: father MI at 45, brother stent at 47. Diet poor, irregular lifestyle. No smoking, occasional alcohol. Exam: Height 180, weight 85 (BMI 26), BP 130/80. No xanthomas or corneal arcus. Labs: TC 310, LDL-C 230, HDL-C 45, TG 175, Lp(a) 40 (N<30). Imaging: ECG normal, echo EF 65%. Carotid US-IMT 0.9 mm, no plaque.

Questions:

How should FH be evaluated with LDL >190 mg/dL?

According to DLCN criteria, what is the FH probability?

What is the treatment approach in primary hypercholesterolemia?

How should drug choice, titration, and follow-up be structured?

Is genetic testing indicated? Should family screening be performed?

Case 23. 64-year-old Male with Statin-Induced Hepatotoxicity

History: CAD with stents ×3 yrs ago, HTN ×10 yrs, DM ×5 yrs. On rosuvastatin 10 mg, but LFT elevation noted, dose reduced to 5 mg. Persistent enzyme rise. No alcohol. Exam: Height 172, weight 82 (BMI 27.7), BP 135/80. Labs: On 10 mg: LDL-C 110, AST 85, ALT 120. On 5 mg: LDL-C 125, AST 100, ALT 150. US: hepatosteatosis grade 1-2. Imaging: ECG-T wave inversions. Echo EF 55%, mild anterior hypokinesia.

Questions:

How should statin-induced liver enzyme elevation be managed?

How is statin-related hepatotoxicity defined, classified, and treated?

What is the management when ALT >3× ULN?

Should statin be stopped, reduced, or switched?

What are alternative lipid-lowering options?

Domain E. Special Patient Groups

Case 24. 62-year-old Female with Diabetic Dyslipidemia and Target Organ Damage

History: 15-year history of type 2 diabetes mellitus with complications: retinopathy, nephropathy (microalbuminuria), and neuropathy. Hypertension ×10 yrs. On insulin glargine, insulin lispro, metformin, amlodipine, irbesartan. No smoking or alcohol. FHx: mother died at 70 from diabetes and CVD, father alive with CVD. Exam: Height 160 cm, weight 80 (BMI 31.2), waist 98 cm, BP 150/90, HR 84. 1+ edema in legs, neuropathy signs. Labs: TC 220, LDL-C 130, HDL-C 38, TG 260, HbA1c 8.2, eGFR 58, microalbuminuria 150 mg/g. Imaging: Echo EF 60% with concentric LVH, mild MR/AR. Carotid plaques 30-40%.

Questions:

How would you classify her CV risk given multiple target organ damage and 3 risk factors?

What are lipid targets and treatment approach in diabetic dyslipidemia?

Which statin and dose should be used initially?

When should combination therapy be considered?

How should triglycerides and HDL be managed in diabetic dyslipidemia?

Case 25. 70-year-old Male on Hemodialysis with Multimorbidity

History: T2DM ×20 yrs, HTN ×15 yrs, CKD ×5 yrs, on hemodialysis ×2 yrs. Prior NSTEMI and CABG ×3 yrs ago. Medications: aspirin, metoprolol, amlodipine, epoetin, phosphate binders, calcitriol. No smoking or alcohol. Exam: Height 170 cm, weight 65 (BMI 22.5), BP 120/70, HR 68. Sternotomy scar, AV fistula, 2+ leg edema. Labs: TC 200, LDL-C 125, HDL-C 35, TG 200, Cr 6.8, eGFR <15, PTH 480, Alb 3.4. Imaging: Echo EF 50%, LVH, inferior hypokinesia, moderate MR.

Questions:

Is statin therapy indicated in end-stage renal disease on hemodialysis?

What is the efficacy and safety of statins in CKD?

Do statins reduce CV events/mortality in dialysis patients?

Which statins can be used without dose adjustment in renal failure?

What drug interactions should be considered in dialysis patients?

Case 26. 58-year-old Male with Acute Coronary Syndrome and Dyslipidemia

History: Presents with chest pain ×2 hrs, diagnosed with NSTEMI. HTN ×10 yrs, T2DM ×5 yrs but nonadherent. Smoker ×25 yrs. Family history positive. On telmisartan, metformin irregularly. Exam: Height 175, weight 88 (BMI 28.7), BP 160/95, HR 95. S3 gallop, basal rales. Labs: Troponin I 8.5, CK-MB 28, TC 235, LDL-C 155, HDL-C 38, TG 210, HbA1c 8.5. Imaging: ECG ST depression V1-V4. Echo EF 45%, anterior wall hypokinesia. Angiography: LAD 90%, Cx 70%, RCA 50% → PCI with stents.

Questions:

What is the optimal early lipid-lowering therapy in ACS?

When should high-intensity statin be started and at what dose?

What are LDL-C goals post-ACS?

When should PCSK9 inhibitors be considered in ACS patients?

How should lipid profile be monitored after ACS?

Case 27. 52-year-old Female with Chronic Liver Disease and Dyslipidemia

History: Chronic hepatitis C → Child-Pugh A cirrhosis. Antiviral therapy completed, viral load negative. HTN ×10 yrs. On olmesartan. Occasional abdominal discomfort. No smoking or alcohol. FHx: mother MI at 68. Exam: Height 165, weight 70 (BMI 25.7), BP 130/80, HR 78. Mild hepatomegaly, no ascites. Labs: TC 220, LDL-C 140, HDL-C 45, TG 175, AST 55, ALT 65, ALP 110, GGT 90, Bilirubin 1.8, Albumin 3.6, INR 1.3, Plt 120k. Imaging: Abdominal US + Fibroscan consistent with cirrhosis (LS 16 kPa). Echo: EF 60%, mild MR.

Questions:

How should dyslipidemia be treated in patients with chronic liver disease?

Is statin therapy safe in compensated cirrhosis?

What are contraindications for statins in liver disease?

Which statins are safer in cirrhosis?

How should liver function be monitored during therapy?

What are non-statin lipid-lowering options in cirrhosis?

Case 28. 75-year-old Female with Stroke, CKD, and CAD

History: Prior ischemic stroke ×2 yrs ago, HTN ×25 yrs, T2DM ×15 yrs, CKD stage 3b, CAD with PCI ×5 yrs ago. Medications: aspirin, clopidogrel, ramipril, amlodipine, metoprolol, furosemide, metformin, insulin, levothyroxine, alendronate. No smoking or alcohol. Exam: Height 160, weight 62 (BMI 24.2), BP 145/85, HR 64. Residual mild right-hand weakness, 1+ edema. Labs: TC 210, LDL-C 135, HDL-C 40, TG 175, HbA1c 7.2, eGFR 45. Imaging: ECG bradycardia, LVH. Echo EF 55%, lateral hypokinesia, moderate MR. Carotid plaques 30-40%.

Questions:

What is her CV risk category given advanced age + multiple comorbidities?

What should her LDL-C target be?

How should lipid therapy be managed in elderly high-risk patients?

What is known about statin efficacy/safety in >75 yrs?

What drug-drug interactions should be considered in polypharmacy?

How should dose adjustment be done with CKD?

Case 29. 67-year-old Male Post-PCI on High-Intensity Statin

History: UA 3 months ago → PCI with stents in LAD and Cx. HTN ×10 yrs, T2DM ×5 yrs. On aspirin, ticagrelor, rosuvastatin 40, bisoprolol, perindopril, metformin. No smoking/alcohol. Exam: Height 170, weight 75 (BMI 26), BP 125/75, HR 62. Labs: TC 125, LDL-C 68, HDL-C 42, TG 120, HbA1c 6.5. Imaging: ECG old T wave inversions. Echo EF 55%, mild hypokinesia.

Questions:

Does LDL-C 68 mg/dL meet current very-high-risk targets?

What are LDL-C goals in very high-risk secondary prevention?

Should statin intensity be reduced if LDL <70?

What is the recommended duration of statin therapy in secondary prevention?

How should lipid follow-up be structured post-PCI?

Case 30. 55-year-old Male with HIV on ART and Dyslipidemia

History: HIV ×8 yrs, on ART with protease inhibitors (ritonavir, atazanavir) + integrase inhibitor (raltegravir). Hypertension ×5 yrs. No CVD. On losartan. Smoker ×15 yrs. No alcohol. Has lipodystrophy (facial lipoatrophy, central fat accumulation). Exam: Height 172, weight 78 (BMI 26.4), waist 94, BP 135/85, HR 80. Labs: TC 240, LDL-C 150, HDL-C 35, TG 250, HbA1c 5.9, HIV RNA undetectable, CD4 550. Imaging: ECG and echo normal.

Questions:

How should dyslipidemia be managed in HIV patients on ART?

What are ART-related effects on lipid profile?

Which statins are safe with protease inhibitors?

How should CV risk be assessed in HIV patients?

Are LDL-C targets different in this population?

Which statin and dose would be appropriate?

Appendix B: Standardized evaluation checklist and scoring rubrics

Section 1: Response Correctness Criteria

LDL-C Target Values (2019 ESC/EAS Guidelines)

Very High Risk: <55 mg/dL and ≥50% reduction from baseline

High Risk: <70 mg/dL and ≥50% reduction from baseline

Moderate Risk: <100 mg/dL

Low Risk: <115 mg/dL

Risk Assessment Tools

Age <70 years: SCORE2

Age ≥70 years: SCORE2-OP

Diabetes: SCORE2-Diabetes

Risk Categories

Very High Risk

Documented ASCVD

SCORE2/SCORE2-OP ≥10%

Diabetes with target organ damage or ≥3 major risk factors

eGFR <30 mL/min/1.73 m²

High Risk

SCORE2/SCORE2-OP 5-9.9%

Markedly elevated single risk factor (TC >310 mg/dL or LDL-C >190 mg/dL)

Diabetes without target organ damage, duration ≥10 years

eGFR 30-59 mL/min/1.73 m²

Section 2: Domain-Specific Scoring Rubrics

Domain 1: Guideline Compliance Accuracy (0-3 points)

3 (High Adherence): Correct risk category, appropriate LDL-C target, evidence-based therapeutic sequence, correct dosing

2 (Partial Compliance): Minor deviations, correct risk assessment but suboptimal targets, appropriate drugs but incorrect dosing

1 (Poor Compliance): Major deviations, incorrect stratification, inappropriate choices

0 (Critical Errors): Completely incorrect, risk of harm, fundamental misunderstanding

Domain 2: Clinical Comprehensiveness (0-3 points)

3 (Comprehensive): All relevant factors addressed, comorbidities/interactions considered, lifestyle advice included, patient-specific adjustments

2 (Adequate): Major aspects covered, minor omissions, some comorbidity consideration

1 (Incomplete): Important factors overlooked, limited patient context, missing key management steps

0 (Inadequate): No consideration of critical issues, oversimplified approach

Domain 3: Practical Implementation (0-3 points)

3 (Directly Applicable): Clear, actionable, with follow-up plan, considers resources, realistic timeline

2 (Mostly Applicable): Practical overall, needs small modifications, adequate follow-up

1 (Limited Applicability): Requires significant modification, unclear steps, weak guidance

0 (Impractical): Unrealistic, no implementation pathway

Domain 4: Communication Effectiveness (0-3 points)

3 (Optimal): Clear, logical, proper terminology, complete explanations, patient-centered when needed

2 (Good): Generally clear, minor structural issues, explanations adequate

1 (Suboptimal): Unclear/confusing, poorly organized, incomplete explanations

0 (Poor): Incomprehensible, contradictory, missing key information

Section 3: Composite Score Interpretation

Good Response: ≥10/12 points (≥83%)

Acceptable Response: 6-9/12 points (50-75%)

Critical Errors: ≤5/12 points (≤42%)

Section 4: Error Classification

Wrong LDL-C Target: Outdated or incorrect thresholds used

Risk Miscalculation: SCORE2/SCORE2-OP applied incorrectly

Wrong Drug Sequence: Skipping statin → ezetimibe → PCSK9i sequence

Dose Errors: Inappropriate statin intensity or dosing

Special Population Errors: Not adjusting for CKD, elderly, pregnancy, HIV therapy

Section 5: Expected Responses by Case Type

Very High-Risk Patients

LDL-C target: <55 mg/dL

High-intensity statin (atorvastatin 40-80 mg or rosuvastatin 20-40 mg)

Add ezetimibe if target unmet

Consider PCSK9 inhibitor if still above target

High-Risk Patients

LDL-C target: <70 mg/dL

High-intensity statin preferred

Combination therapy as needed

Special Populations

CKD: Dose adjustment for statins with renal clearance

Elderly >75 years: Start low, titrate, monitor

Pregnancy: Statins contraindicated

HIV on ART: Avoid simvastatin with protease inhibitors

Section 6: Standardized Prompts for AI Models

"Please provide comprehensive dyslipidemia management recommendations for the following clinical case, including cardiovascular risk assessment and category, specific lipid targets, lifestyle modification recommendations, pharmacological therapy selection and dosing, follow-up and monitoring plan. Base your recommendations on current ESC/EAS guidelines."
